# Comparing the Dosing Period in Package Inserts of Antimicrobial Agents Between Japan and the United States

**DOI:** 10.7759/cureus.38266

**Published:** 2023-04-28

**Authors:** Tetsuta Nishigaki, Hideaki Kato, Yasutaka Sakamoto, Tomoyo Suzuki, Mirei Kaneko, Kazuo Ide, Nakaba Okamura, Taiichi Suzuki, Hirofumi Koike, Yukiko Sahashi

**Affiliations:** 1 Pharmaceutical Department, Yokohama City University Hospital, Yokohama, JPN; 2 Infection Prevention and Control Department, Yokohama City University Hospital, Yokohama, JPN; 3 School of Pharmacy, Kitasato University, Tokyo, JPN

**Keywords:** antibiotic prescription, behavior change, duration of treament, iv drug, continued drug use, antimicrobial stewardship program, antimicrobial drugs (amds), dosing period, package insert

## Abstract

*Introduction*: The duration of antimicrobial therapy is a critical evaluation index of antimicrobial stewardship (AS). The inclusion of the dosing period on package inserts provides a strong reason for clinical intervention by pharmacists in cases where physicians prescribe inappropriate dosing periods. This study investigated differences in the description of dosing periods in antimicrobial package inserts between Japan and the U.S.

*Methods*: We conducted a survey comparing differences in the dosing period of oral and injectable antimicrobials approved and marketed in Japan and the U.S. as of May 1, 2021. The Fisher exact test was used to compare the presence or absence of a description of the dosing period on the package insert between these two countries.

*Results*: We evaluated 69 antimicrobial agents, of which 34 were oral; and 35 were injectable agents. In Japan, 20 (29.0%) of the antimicrobials had package inserts stating the dosing periods, compared with 58 (84.1%) in the U.S. (p < 0.001).

*Conclusions*: It was found that the information on the duration of administration was missing from the package insert in Japan compared to the U.S. Lack of information on the duration of administration may lead to long-term administration by the treating physician and also make it difficult for the pharmacist to inquire about the administration. It is expected that the inclusion of scientifically-based dosing periods in all package inserts will promote AS among physicians and pharmacists who are not specialists in infectious disease therapy.

## Introduction

Worldwide measures to prepare for the threat of drug-resistant bacteria are being implemented based on the WHO’s Global Action Plan [1]. In Japan, since 2016, measures against drug-resistant bacteria have been taken in six areas by the National Action Plan on Antimicrobial Resistance (AMR). The outcome indices for these measures are the proportion of specified antimicrobial-resistant bacteria and the use of antimicrobials [2]. The aims of antimicrobial stewardship (AS) are to determine indications for antimicrobials and optimal antimicrobial regimens to improve patients' outcomes and minimize the adverse events caused by antimicrobial use [3].

In Japan, the package insert was established by Articles 52, 54, and 68-2 of the Act on Securing Quality, Efficacy, and Safety of Products, Including Pharmaceuticals and Medical Devices (commonly known as the Pharmaceutical and Medical Device [PMD] Act]) [4]. Among the many sources of drug information, the package insert is the only document established by law that is attached to any drug. Based on the order of the Ministry of Health, Labour, and Welfare (MHLW) [5], the PMD Act states that dose, administration, and other necessary care for the use and handling of information are required. While there is no regulation for treatment duration on package insert under the PMD Act. As a result, information on the dosing period is not always included in the package inserts of antimicrobial agents in Japan, and physicians like to administer longer durations than generally recommended [6].

In the U.S., Federal Regulation Section 201.100 (Prescription of drugs for human use) stipulates the package insert must include information about the indications, effects, dose, routes, methods, frequency, and dosing period. Additionally, Federal Regulation Section 314.50 (content and format of a new drug application) states a proposed package insert must be submitted for new drug applications. In the Japanese antimicrobial stewardship guidelines, it was suggested that the preceding U.S. practices be investigated, and we considered a comparison between the U.S. and Japan [7]. So far, there are limited reports comparing the dosing period stated in antimicrobial package inserts between Japan and the U.S. from the perspective of AS. We considered that the difference in the description of the treatment duration in the package insert might affect the appropriate use of antimicrobial agents and investigated the difference in the description of the dosing period in the package insert.

## Materials and methods

Analyzed antimicrobial agents

We investigated medical antimicrobial agents for oral and injectable use that were approved and marketed in both Japan and the U.S. as of May 1, 2021. We extracted the data for Japan using the drug database published on the Health Insurance Claims Review and Reimbursement Services website [8]. We obtained the package inserts of the antimicrobial agents in Japan from the Pharmaceuticals and Medical Devices Agency website [9] and those in the U.S. from the Drugs@FDA [10] or DailyMed websites [11]. In cases where both original and generic versions of the same drug were available, we used the package insert of the original drug for our survey. We excluded antimicrobials from our study if an agent with the same ingredients was not approved and marketed in the U.S. or if the antimicrobial did not include bacterial infections as one of its indications for use. Only the adult formulation was included if oral medications included both an adult and a pediatric formulation. If there were multiple forms of an oral drug with the same ingredients, only one form was included in the dosage, and administration was the same (in order of priority: tablets, capsules, dispersions, and liquids). If there were multiple indications, the duration of administration was considered to be stated if it was stated for at least one of the indications. 

Statistical analysis

We compared the presence or absence of a description of the dosing period on the package insert between Japan and the U.S. using the Fisher exact test. A two-tailed p-value of < 0.05 was considered statistically significant. All statistical analyses were performed using JMP Pro v15 software (SAS Institute Japan, Tokyo, Japan).

## Results

**Figure 1:**
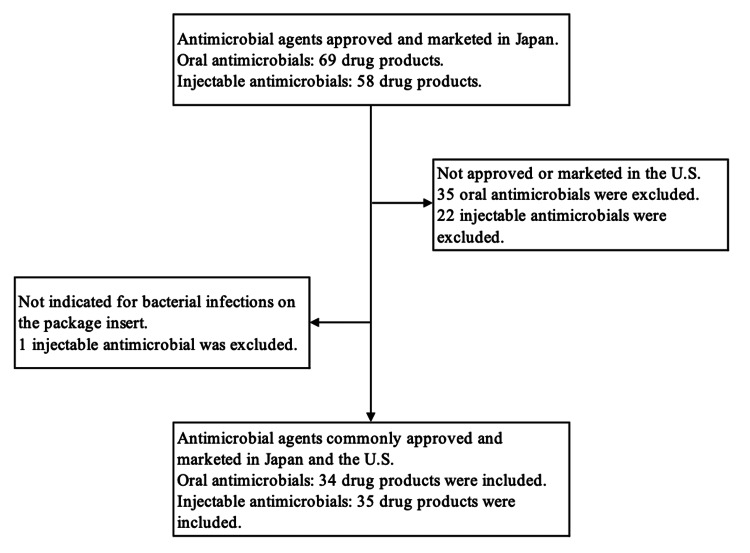
Antimicrobial agents included and excluded in our study

Of the 69 antimicrobial agents that were approved and marketed in both Japan and the U.S. as of May 1, 2021, 34 were oral, and 35 were injectable drugs. Among the oral antimicrobials, nine (26.5%) were antituberculous agents (Figure 1, Table 1, and Table 2). β-lactams accounted for 14 (40.0%) of the injectable drugs. 

**Table 1:** Oral antimicrobials analyzed in this study, which were approved and marketed in both Japan and the U.S. (as of May 1, 2021).

Oral antimicrobials (34 drug products)	
Antituberculous agents	Macrolides
Bedaquiline	Azithromycin (250mg, 500mg Tablet)
Cycloserine	Clarithromycin
Ethambutol	Erythromycin
Ethionamide	Fidaxomicin
Isoniazid	
p-aminosalicylic acid	Tetracyclines
Pyrazinamide	Doxycycline
Rifabutin	Minocycline
Rifampicin	Tetracycline
Cephems	Oxazolidinones
Cefaclor	Linezolid
Cefdinir	Tedizolid
Cefixime	
Cefpodoxime	Penicillins
Cefuroxime	Amoxicillin
Cephalexin	Clavulanate/Amoxicillin
Fluoroquinolones	Others
Ciprofloxacin	Clindamycin
Levofloxacin	Metronidazole
Moxifloxacin	Sulfamethoxazole-Trimethoprim
Ofloxacin	Vancomycin

**Table 2:** Injectable antimicrobials analyzed in this study were approved and marketed in both Japan and the U.S. (as of May 1, 2021).

Injectable antimicrobials (35 drug products)	
Cephems	Fluoroquinolones
Cefazolin	Ciprofloxacin
Cefepime	Levofloxacin
Cefotaxime	
Ceftazidime	Lincomycins
Ceftriaxone	Clindamycin
Tazobactam/Ceftolozane	Lincomycin
Penicillins	Macrolides
Ampicillin	Azithromycin
Benzylpenicillin	Erythromycin
Piperacillin	
Sulbactam/Ampicillin	Oxazolidinones
Tazobactam/Piperacillin	Linezolid
	Tedizolid
Carbapenems	
Doripenem	Others
Imipenem/Cilastatin	Aztreonam
Meropenem	Chloramphenicol
	Colistin
Aminoglycosides	Daptomycin
Amikacin	Isoniazid
Gentamicin	Metronidazole
Streptomycin	Minocycline
Tobramycin	Tigecycline
	Vancomycin

Comparisons of all antimicrobial agents (oral and injectable) revealed that 20 (29.0%) and 58 (84.1%) of the drug products included in this study listed the dosing period in the package insert in Japan and the U.S., respectively (*p* < 0.001). In a comparison of oral antimicrobials only, 14 (41.2%) and 29 (85.3%) of the drug products included in this study listed the dosing period in Japan and the U.S., respectively (p < 0.001). A comparison of the injectable antimicrobials only revealed that six (17.1%) and 29 (82.9%) of the drug products included in this study listed the dosing period in Japan and the U.S., respectively (*p* < 0.001) (Table 3).

The indications and the dosing period indicated on the package inserts for the antimicrobial agents are in Table 4 (Japan) and Table 5 (the U.S.).

**Table 3:** Comparison of the presence of dosing periods on package inserts between Japan and the U.S. The number represented the number of drugs for which the duration of administration was listed for at least one indication in the package inserts. The percentages were calculated by the number of drugs included in this study. The data were statistically compared by Fisher's exact test. * statistically significant

	Japan	The U.S.	P-value
Oral and injectable antimicrobial agents (n=69)			
Dosing period is described in the package insert (n, (%))	20 (29.0)	58 (84.1)	< 0.001 *
Oral antimicrobial agents (n=34)			
Dosing period is described in the package insert (n, (%))	14 (41.2)	29 (85.3)	< 0.001 *
Injectable antimicrobial agents (n=35)			
Dosing period is described in the package insert (n, (%))	6 (17.1)	29 (82.9)	< 0.001 *

**Table 4:** Antimicrobial agents for which the dosing period is described in the Japanese package insert. MAC: Mycobacterium avium complex; MRSA: Methicillin-resistant Staphylococcus aureus; CAP: Community-acquired pneumonia; HAP: Hospital-acquired pneumonia

Antimicrobial agents	Dosage form	Type of infection (duration)
Fluoroquinolones		
Ciprofloxacin	Oral medicine	anthrax (60 days)
Levofloxacin	Injection drug	typhoid fever, paratyphoid fever (14 days); anthrax (60 days)
Levofloxacin	Oral medicine	typhoid fever, paratyphoid fever (14 days); anthrax (60 days)
Moxifloxacin	Oral medicine	cutaneous infections, pharyngo-laryngitis, tonsillitis, acute bronchitis, secondary infection in chronic respiratory lesions (within 7 days); pneumonia, sinusitis (within 10 days)
Ofloxacin	Oral medicine	typhoid fever, paratyphoid fever (14 days)
Macrolides		
Azithromycin	Oral medicine	chlamydial urethritis, chlamydial cervicitis (1 day); deep-seated skin infections, lymphangitis and lymphadenitis, pharyngo-laryngitis, tonsillitis, acute bronchitis, pneumonia, lung abscess, secondary infection in chronic respiratory lesions, sinusitis, periodontitis, pericoronitis, jaw inflammation (3 days)
Clarithromycin	Oral medicine	*Helicobacter pylori* infection [with other drugs] (7 days); chlamydial infections (2 to 3 weeks after symptoms improve); legionella pneumonia (2 to 3 weeks after treatment is completed); pulmonary MAC disease in immunocompromised hosts (more than 1 year)
Fidaxomicin	Oral medicine	infectious enterocolitis (10 days)
Tetracyclines		
Doxycycline	Oral medicine	anthrax (60 days)
Minocycline	Oral medicine	anthrax (60 days)
Minocycline	Injection drug	anthrax (60 days)
Oxazolidinones		
Linezolid	Oral medicine	sepsis, deep-seated skin infections, chronic pyoderma, secondary infection in trauma, burns, and surgical wounds, pneumonia, various infectious diseases caused by MRSA (within 29 days)
Linezolid	Injection drug	sepsis, deep-seated skin infections, chronic pyoderma, secondary infection in trauma, burns, and surgical wounds, pneumonia, various infectious diseases caused by MRSA (within 29 days)
Penicillins		
Amoxicillin	Oral medicine	*Helicobacter pylori* infection [with other drugs] (7 days)
Tazobactam/Piperacillin	Injection drug	pyelonephritis, complicated cystitis (5 days); deep-seated skin infections, secondary infection of erosions and ulcers, CAP, peritonitis, abdominal abscess, cholecystitis, cholangitis, febrile neutropenia, pyelonephritis in children, complicated cystitis in children ( 14 days); sepsis, HAP (21 days)
Others		
Bedaquiline	Oral medicine	multidrug resistance pulmonary tuberculosis [with other antituberculous agents] (6 months)
Cefepime	Injection drug	sepsis, deep-seated skin infections, secondary infection in trauma, burns, and surgical wounds, periproctal abscess, tonsillitis, pneumonia, lung abscess, secondary infection in chronic respiratory lesions, complicated cystitis, pyelonephritis, prostatitis. peritonitis, abdominal abscess, cholecystitis, cholangitis, intrauterine infection, parametritis, otitis media, sinusitis (within 14 days)
Metronidazole	Oral medicine	infectious enterocolitis (10 to 14 days); bacterial vaginosis, *Helicobacter pylori* infection [with other drugs] (7 days)
Tigecycline	Injection drug	deep-seated skin infections, chronic pyoderma, secondary infections of external wounds, burns and surgical wounds, secondary infections of erosions and ulcers, peritonitis, intraabdominal abscess, cholecystitis (5 to 14 days)
Vancomycin	Oral medicine	(in the case that there is no sign of symptom improvement, such as diarrhea, abdominal pain, fever, etc.) infectious enterocolitis (7 to 10 days)

**Table 5:** Antimicrobial agents for which the dosing period is described in the U.S. package insert. CAP: community acquired pneumonia; SSTI: skin and soft tissue infections; UTI: urinary tract infections; CNSI: central nervous system infections; HAP: hospital-acquired pneumonia; VAP: ventilator-associated pneumonia; QD: quaque die; QID: quater in die; BID: bis in die

Antimicrobial agents	Dosage form	Duration and type of infection
Cephems		
Cefaclor	Oral medicine	β-hemolytic streptococcal infections (10 days)
Cefazolin	Injection drug	perioperative prophylaxis (Within a 24 hour period after the surgical procedure); in surgery where the occurrence of infection may be particularly devastating (e.g., open-heart surgery and prosthetic arthroplasty) (3 to 5 days following the completion of surgery)
Cefdinir	Oral medicine	acute exacerbation of chronic bronchitis [300mg q12hr], pharyngitis/tonsillitis [300mg q12hr] (5 to 10 days); CAP, acute exacerbation of chronic bronchitis [600mg q24hr], acute maxillary sinusitis, pharyngitis/tonsillitis [600mg q24hr], uncomplicated SSTI (10 days)
Cefepime	Injection drug	empiric therapy for febrile neutropenia [or until resolution of neutropenia. In patients whose fever resolves but who remain neutropenic for more than 7 days] (7 days); mild to moderate uncomplicated or complicated UTI, complicated intraabdominal infection (used in combination with metronidazole) (7 to 10 days); moderate to severe pneumonia, severe uncomplicated or complicated UTI, moderate to severe uncomplicated SSTI (10 days)
Cefixime	Oral medicine	infections due to *Streptococcus pyogenes* (at least 10 days)
Cefotaxime	Injection drug	prevention of postoperative infection (1 day); bacteremia, septicemia, lower respiratory tract infections, bone and/or joint infections, genitourinary infections, gynecologic infections, CNSI, SSTI, intraabdominal infections (minimum of 48 to 72 hours after the patient defervesces or after evidence of bacterial eradication has been obtained); infection caused by group A beta-hemolytic streptococci (minimum of 10 days)
Cefpodoxime	Oral medicine	uncomplicated gonorrhea, rectal gonococcal infections in women (1 day); pharyngitis and/or tonsillitis (5 to 10 days); uncomplicated UTI (7 days); SSTI (7 to 14 days); acute bacterial exacerbation of chronic bronchitis, acute maxillary sinusitis (10 days); CAP (14 days)
Ceftazidime	Injection drug	bacterial septicemia, lower respiratory tract infections, bone and joint infections, gynecologic infections, CNSI, UTI, both complicated and uncomplicated SSTI, intraabdominal infections (Generally, therapy should be continued for 2 days after the signs and symptoms of infection have disappeared)
Ceftriaxone	Injection drug	surgical prophylaxis (1 day); bacterial septicemia, lower respiratory tract infections, bone and joint infections, acute bacterial otitis media, uncomplicated gonorrhea, meningitis (4 to 14 days, in complicated infections, longer therapy may be required); meningitis in pediatric patients (7 to 14 days); infections caused by *Streptococcus pyogenes* (at least 10 days)
Cefuroxime	Oral medicine	uncomplicated gonorrhea (1 day); uncomplicated UTI (7 to 10 days); pharyngitis/tonsillitis (mild to moderate), acute bacterial maxillary sinusitis (mild to moderate), acute bacterial exacerbation of chronic bronchitis (mild to moderate), uncomplicated SSTI, acute bacterial otitis media in pediatric patients younger than 13 years (10 days); early Lyme disease (20 days)
Cephalexin	Oral medicine	usual duration of therapy (7 to 14 days)
Tazobactam/Ceftolozane	Injection drug	complicated UTI (7 days); complicated intraabdominal infections (4 to 14 days); HAP, VAP (8 to 14 days)
Penicillins		
Amoxicillin	Oral medicine	any infections caused by *Streptococcus pyogenes* (at least 10 days); *Helicobacter pylori* infection [with other drugs] (14 days)
Ampicillin	Injection drug	endocarditis, septicemia, respiratory tract infections, gastrointestinal infections, bacterial meningitis, UTI (Minimum of 48 to 72 hours beyond the time that the patient becomes asymptomatic or evidence of bacterial eradication has been obtained); any infection caused by group A beta-hemolytic streptococci (minimum of 10 days)
Benzylpenicillin	Injection drug	most acute infections (at least 48 to 72 hours after the patient becomes asymptomatic); all infections due to group A beta-hemolytic streptococci (at least 10 days); gonococcal arthritis in pediatric patients (7 to 10 days); meningitis caused by pneumococcus and meningococcus in pediatric patients (7 to 14 days); diphtheria (adjunctive therapy to antitoxin and for the prevention of the carrier state) (10 to 12 days); gonococcal meningitis in pediatric patients (10 to 14 days); syphilis [neurosyphilis] (10 to 14 days [many experts recommend additional therapy with benzathine penicillin G 2.4 MU intramuscular weekly for 3 doses after completion of intravenous therapy]); pasteurella infections, meningitis caused by listeria (2 weeks); haverhill fever, rat-bite fever (3 to 4 weeks); endocarditis caused by listeria, gonococcal endocarditis in pediatric patients (4 weeks); erysipelothrix endocarditis (4 to 6 weeks)
Clavulanate/Amoxicillin	Oral medicine	acute otitis media (10 days)
Piperacillin	Injection drug	prophylactic use in surgery (should be stopped within 24 hours); most acute infections (at least 48 to 72 hours after the patient becomes asymptomatic); *Streptococcus pyogenes* infections (at least 10 days); gynecologic infections (3 to 10 days [the duration should be guided by the patient's clinical and bacteriological progress]); septicemia, lower respiratory tract infections, bone and joint Infections, uncomplicated gonococcal urethritis, UTI, SSTI, Intraabdominal infections (7 to 10 days [the duration should be guided by the patient's clinical and bacteriological progress])
Sulbactam/Ampicillin	Injection drug	SSTI in pediatric patients 1 year of age or older (should not routinely exceed 14 days [course of intravenous therapy])
Tazobactam/Piperacillin	Injection drug	intraabdominal infections, SSTI, female pelvic infections, CAP (7 to 10 days); HAP (7 to 14 days)
Fluoroquinolones		
Ciprofloxacin	Injection drug	SSTI, complicated intraabdominal infections, empirical therapy in febrile neutropenic, lower respiratory tract infection, UTI (7 to 14 days); acute sinusitis (10 days); HAP (10 to 14 days); plague in pediatric, complicated UTI or pyelonephritis in pediatric (10 to 21 days); plague in adults (14 days); chronic bacterial prostatitis (28 days); bone and joint infections (4 to 8 weeks); inhalational anthrax [post-exposure] (60 days)
Ciprofloxacin	Oral medicine	uncomplicated gonorrhea (1 day); acute uncomplicated cystitis (3 days); infectious diarrhea (5 to 7 days); SSTI, complicated intraabdominal infection, lower respiratory tract infections, UTI (7 to 14 days); typhoid fever, acute sinusitis (10 days); plague (14 days); chronic bacterial prostatitis (28 days); inhalational anthrax [postexposure] (60 days); bone and joint infections (4 to 8 weeks)
Levofloxacin	Injection drug	uncomplicated UTI (3 days); CAP [750mg QD], complicated UTI or acute pyelonephritis [750mg QD], acute bacterial sinusitis [750mg QD] (5 days); acute bacterial exacerbation of chronic bronchitis (7days); uncomplicated SSTI (7 to 10 days); HAP, CAP [500mg QD], complicated SSTI (7 to 14 days); complicated UTI or acute pyelonephritis [250mg QD] (10 days); plague, acute bacterial sinusitis [500mg QD] (10 to 14 days); chronic bacterial prostatitis (28 days); inhalational anthrax [post-exposure] (60 days)
Levofloxacin	Oral medicine	uncomplicated UTI (3 days); CAP [750mg QD], complicated UTI or acute pyelonephritis [750mg QD], acute bacterial sinusitis [750mg QD] (5 days); acute bacterial exacerbation of chronic bronchitis (7 days); uncomplicated SSTI (7 to 10 days); HAP, CAP [500mg QD], complicated SSTI (7 to 14 days); complicated UTI or acute pyelonephritis [250mg QD] (10 days); plague, acute bacterial sinusitis [500mg QD] (10 to 14 days); chronic bacterial prostatitis (28 days); inhalational anthrax [post-exposure] (60 days)
Moxifloxacin	Oral medicine	acute bacterial exacerbation of chronic bronchitis (5 days); complicated intraabdominal infections (5 to 14 days); uncomplicated SSTI (7 days); CAP (7 to 14 days); complicated SSTI (up to 21 days); acute bacterial sinusitis (10 days); plague (10 to 14 days)
Ofloxacin	Oral medicine	acute uncomplicated urethral and cervical gonorrhea (1 day); uncomplicated cystitis due to *Escherichia coli* or *Klebsiella pneumoniae* (3 days); nongonococcal cervicitis/urethritis due to *Chlamydia trachomatis*, mixed infection of the urethra and cervix due to *C. trachomatis* and *Neisseria gonorrhoeae*, uncomplicated cystitis due to approved pathogens (other than *E. coli* or *K. pneumoniae*) (7 days); acute bacterial exacerbation of chronic bronchitis, CAP, uncomplicated SSTI, complicated UTI (10 days); acute pelvic inflammatory disease (10 to 14 days); prostatitis due to *E. coli *(6 weeks)
Macrolides		
Azithromycin	Injection drug	pelvic inflammatory disease (7 days); CAP (7 to 10 days)
Azithromycin (250mg, 500mg Tablet)	Oral medicine	genital ulcer disease [chancroid], non-gonococcal urethritis and cervicitis, gonococcal urethritis and cervicitis (1 day); CAP [mild severity], pharyngitis/tonsillitis [second-line therapy], uncomplicated SSTI, acute exacerbation of chronic bronchitis [mild to moderate, 500 mg as a single dose on day 1, followed by 250 mg once daily], acute otitis media in 6 months of age and older 3 days: acute exacerbation of chronic bronchitis [mild to moderate, 500 mg once daily for 3 days], acute bacterial sinusitis (2 to 5 days)
Clarithromycin	Oral medicine	acute maxillary sinusitis caused by *Haemophilus influenzae* (7 days); acute bacterial exacerbation of chronic bronchitis, CAP, uncomplicated SSTI (7 to 14 days); pharyngitis/tonsillitis (10 days); *Helicobacter pylori* eradication [with amoxicillin and omeprazole or lansoprazole] (10 to 14 days); acute maxillary sinusitis, *Helicobacter pylori* eradication [with omeprazole] (14 days)
Erythromycin	Injection drug	group A beta-hemolytic streptococcal infections of the upper respiratory tract in penicillin-allergic patients, acute pelvic inflammatory disease caused by *Neisseria gonorrhoeae* in patients hypersensitive to penicillin (10 days)
Erythromycin	Oral medicine	urogenital infection during pregnancy due to *Chlamydia trachomatis* [500mg QID], adults with uncomplicated urethral, endocervical, or rectal infections caused by *C. trachomatis* [when tetracycline is contraindicated or not tolerated], patients with nongonococcal urethritis caused by *Ureaplasma urealyticum* [when tetracycline is contraindicated or not tolerated] (at least 7 days); streptococcal infections of the upper respiratory tract in children (at least 10 days: ); conjunctivitis of the newborn caused by *C. trachomatis*, urogenital infection during pregnancy due to *C. trachomatis* for women who can not tolerate 500mg QID regimen [500mg BID or 250mg QID] (at least 2 weeks); pneumonia of infancy caused by *C. trachomatis* (at least 3 weeks); pertussis (5 to 14 days); acute pelvic inflammatory disease caused by *Neisseria gonorrhoeae* (10 days); primary syphilis (10 to 15 days)
Fidaxomicin	Oral medicine	*Clostridioides difficile*-associated diarrhea (10 days)
Antituberculous agents		
Bedaquiline	Oral medicine	pulmonary multi-drug resistant tuberculosis (24 weeks)
Isoniazid	Injection drug	pulmonary tuberculosis without HIV infection [with rifampicin and pyrazinamide] (24 weeks); pulmonary tuberculosis without HIV infection [with rifampicin, pyrazinamide and ethambutol or streptomycin] (6 months); extra pulmonary tuberculosis [with other antituberculous agents] (6 to 9 months)
Isoniazid	Oral medicine	pulmonary tuberculosis without HIV infection [with rifampicin and pyrazinamide] (24 weeks); pulmonary tuberculosis without HIV infection [with rifampicin, pyrazinamide and ethambutol or streptomycin] (6 months); extra pulmonary tuberculosis [with other antituberculous agents] (6 to 9 months)
Pyrazinamide	Oral medicine	tuberculosis [initial 2 months of 6-month regimen] (2 months)
Rifampicin	Oral medicine	meningococcal carriers (2 days); tuberculosis (6 months)
Aminoglycosides		
Amikacin	Injection drug	sepsis, respiratory tract infections, osteoarticular infections, CNSI, SSTI, intraabdominal infections, burns and postoperative infections, complicated and recurrent UTI (7 to 10 days)
Gentamicin	Injection drug	sepsis, respiratory tract infections, gastrointestinal infections, CNSI, UTI, skin, bone, and skin structure infections (7 to10 days)
Streptomycin	Injection drug	tularemia (7 to 14 days until the patient is afebrile for 5 to 7 days); plague (minimum of 10 days); streptococcal endocarditis [concomitantly with penicillin] (2 weeks); enterococcal endocarditis [concomitantly with penicillin] (4 weeks); tuberculosis (minimum of 1 year)
Tobramycin	Injection drug	sepsis, lower respiratory tract infections, CNSI, intraabdominal infections, skin, bone, and skin structure infections, complicated UTI (7 to10 days)
Oxazolidinones		
Linezolid	Injection drug	HAP, CAP, complicated SSTI, uncomplicated SSTI (10 to 14 days); vancomycin-resistant *Enterococcus faecium* infections (14 to 28 days)
Linezolid	Oral medicine	HAP, CAP, complicated SSTI, uncomplicated SSTI (10 to 14 days); vancomycin-resistant E*nterococcus faecium* infections (14 to 28 days)
Tedizolid	Injection drug	acute bacterial SSTI (6 days)
Tedizolid	Oral medicine	acute bacterial SSTI (6 days)
Tetracyclines		
Doxycycline	Oral medicine	uncomplicated gonococcal infections in adults [except anorectal infections in men], uncomplicated urethral, endocervical or rectal infections in adults caused by *Chlamydia trachomatis*, nongonococcal urethritis caused by *C. trachomatis* or *Ureaplasma urealyticum* (7 days); acute epididymo-orchitis caused by *Neisseria gonorrhoeae* or *C. trachomatis* (at least 10 days); streptococcal infections (10 days); syphilis-early in patients allergic to penicillin (2 weeks); syphilis of more than one year’s duration in patients allergic to penicillin (4 weeks); inhalational anthrax [post-exposure] (60 days)
Minocycline	Oral medicine	uncomplicated gonococcal infections other than urethritis and anorectal infections in men (minimum 4 days); uncomplicated gonococcal urethritis in men, meningococcal carrier state (5 days); endocervical or rectal infections caused by *Chlamydia trachomatis* or *Ureaplasma urealyticum* (at least 7 days); syphilis (10 to 15 days); *Mycobacterium marinum* infections (6 to 8 weeks)
Tetracycline	Oral medicine	uncomplicated urethral, endocervical or rectal infections caused by *Chlamydia trachomatis* (at least 7days); gonorrhea (7 days); streptococcal infections (10 days); early syphilis in patients allergic to penicillin (15 days); brucellosis endocervical [accompanied by streptomycin] or rectal infections in adults caused by *C. trachomatis* (3 weeks); syphilis of more than one year's duration [except neurosyphilis] in patients allergic to penicillin (30 days)
Others		
Aztreonam	Injection drug	septicemia, lower respiratory tract infections, gynecologic infections, UTI, SSTI, intraabdominal infections (at least 48 hours after the patient becomes asymptomatic or evidence of bacterial eradication has been obtained)
Chloramphenicol	Oral medicine	typhoid fever (8 to 10 days after the patient has become afebrile)
Clindamycin	Injection drug	β-hemolytic streptococcal infection (at least 10 days)
Clindamycin	Oral medicine	β-hemolytic streptococcal infection (at least 10 days)
Daptomycin	Injection drug	complicated SSTI in adults (7 to 14 days); *Staphylococcus aureus* bacteremia in adults (2 to 6 weeks); complicated SSTI in children (up to 14 days); *S. aureus* bacteremia in children (up to 42 days)
Doripenem	Injection drug	complicated UTI (10 days, extended up to 14 days for patients with concurrent bacteremia); complicated intraabdominal infections (5 to 14 days)
Metronidazole	Injection drug	perioperative prophylaxis (1 day); anaerobic infections [however, infections of the bone and joint, lower respiratory tract, and endocardium may require longer treatment] (7 to 10 days)
Metronidazole	Oral medicine	anaerobic infections [however, infections of the bone and joint, lower respiratory tract, and endocardium may require longer treatment] (7 to 10 days)
Sulfamethoxazole-Trimethoprim	Oral medicine	shigellosis, traveler’s diarrhea (5 days); UTI (10 to 14 days); acute otitis media in children (10 days); acute exacerbation of chronic bronchitis (14 days)
Tigecycline	Injection drug	complicated SSTI, complicated intraabdominal infections (5 to 10 days); CAP (7 to 14 days)
Vancomycin	Oral medicine	staphylococcal enterocolitis (7 to 10 days); *Clostridioides difficile*-associated diarrhea (10 days)

## Discussion

Our study is the first report focusing on the absence of information on the dosing period in the package inserts in Japan and comparing the package inserts of the antimicrobial agents in Japan and the U.S. optimizing the dosing period is a critical issue in promoting AS. Our findings showed that the rate of inclusion of the dosing period in the package inserts of antimicrobial agents in Japan was <50% of that of the U.S. Especially the description of the dosing period for infections such as pneumonia and urinary tract infections was low (Table 4). In contrast, there were frequent descriptions of the dosing period for pneumonia and urinary tract infections in the package inserts for the U.S. antimicrobials (Table 5). For example, in the U.S., the package insert for levofloxacin (LVFX) includes descriptions of the dosing period for 12 types of infections. We also found that not all indications are listed in the Japanese package insert (Table 4). It is suggested to list clinical indications for reference by clinicians. Although there are guidelines on infectious disease treatment, the package inserts are attached to all products and are highly accessible. Practical information included in the package inserts may assist the treating physicians in administering antibiotics.

The package insert is the document pharmacists most often refer to when questioning doctors about prescriptions [12]. A questionnaire survey about AS administered to Japanese pharmacists reported that 72.7% of them had been aware of inappropriate antimicrobial use, but only 28.5% had intervened [13]. In the same survey, only 25.1% of pharmacists could collect sufficient information about AS for prescription questions [14]. The lack of information on the dosing period in the package inserts of antimicrobial agents may make it difficult for pharmacists to intervene in cases of the inappropriate duration of antimicrobial therapy. The lack of a dosing period in the package inserts of antimicrobials in Japan may affect the physician's consideration of the duration of antimicrobial therapy. A questionnaire survey reported that 20% of Japanese hospital doctors administer antimicrobials without criteria for the dosing period [14]. It is crucial to ensure appropriate dosing periods by combining prospective audit and feedback, education, and the development of regional and facility-specific guidelines, as well as revising information in package inserts [15]. Enhancing the description of the dosing period in the package inserts is helpful for pharmacists and physicians who are not specialists in administering antibiotics with the correct dosing period. One of the actions of the National Action Plan on AMR is to review the information included in package inserts (e.g., precautions for use) for antimicrobials based on scientific evidence [2]. 

In Japan, there are no reports of extensive surveys on the appropriateness of antimicrobial drug administration, including the dosing period. Multiple interventions are performed simultaneously in many cases of AS, making it difficult to assess the impact of specific interventions [16]. Thus, assessing the outcome of including the dosing period in the antimicrobial package inserts is challenging. However, the package insert is supposed to promote the appropriateness of antimicrobial administration. Kimura et al. reported that physicians in Japan treat patients with Clostridioides difficile infection using metronidazole (median: 10 days, interquartile range: 7-13 days) [17], and the dosing period for enteritis is listed as 10-14 days in the package insert in Japan. The result suggested that including the dosing period in this package, insert may contribute to treatment at the appropriate dosing period. 

Our study had several limitations. First, although we surveyed oral and injectable antimicrobial agents approved and marketed in Japan and the U.S., the indications for antimicrobial agents in the two countries may differ. It should also be noted that no comparisons were made with European countries. Our survey did not compare or evaluate the dosing period listed in the package inserts of antimicrobial agents with the duration recommended in guidelines for various infectious diseases. There are also differences between Japan and the U.S. in dosage. For example, the maximum daily dosage of levofloxacin differs between the US and Japan (750 mg and 500 mg). Although this study focused on the duration of administration, the dosage should also be considered. In addition, how the inclusion of package inserts affects pharmacist intervention and the reduction in prescribing time for physicians was not analyzed in this study. The impact of modifying the package inserts also needs to be analyzed.

## Conclusions

As a conclusion, there was an absence of dosing period information in the package inserts in Japan compared with the U.S. The description of a scientifically based dosing period in the package inserts of antimicrobials in Japan is expected to promote AS among physicians and pharmacists who are not specialists in infectious disease therapy.
